# Cancer testis antigen 55 deficiency attenuates colitis-associated colorectal cancer by inhibiting NF-κB signaling

**DOI:** 10.1038/s41419-019-1537-x

**Published:** 2019-04-03

**Authors:** Huan Zhao, Wen-Ming Pan, Hui-Hui Zhang, Yang Song, Jie Chen, Ying Xiang, Bo Gu, Shang-Ze Li, Run-Lei Du, Xiao-Dong Zhang

**Affiliations:** 1 Hubei Key Laboratory of Cell Homeostasis, College of Life Sciences, Wuhan University, Wuhan, China; 2 Department of Emergency Surgery, Union Hospital, Tongji Medical College, Huazhong University of Science and Technology, Wuhan, China; 3 Laboratory of Molecular Biology, School of Medicine in Hunan Normal University, Changsha, Hunan China; 4 Medical Science Research Center, Zhongnan Hospital of Wuhan University, Wuhan, China

## Abstract

Colitis-associated cancer (CAC), a prototype of inflammation-associated cancer, is one of the most common gastrointestinal tumors. As a potential cancer testis antigen (CT antigen), cancer testis antigen 55 (CT55) is expressed in different tumors and normal testes. However, its role in CAC remains unknown. Here, we identified CT55 as a new potent promoter of CAC. We discovered that Ct55 deficiency alleviated inflammatory responses, decreased cell proliferation and colitis-associated tumorigenesis in an azoxymethane/dextran sulfate sodium (AOM/DSS) mouse model. Mechanistically, CT55 acts as an accelerator of tumor necrosis factor (TNF)-α-induced nuclear factor-κB (NF-κB) signaling. Upon stimulation with TNF-α, CT55 interacts with the IκB kinase (IKK) complex, which increases the phosphorylation of IKKα/β and activates IKK–p65 signaling, while knockout of CT55 blocks IKK–p65 signaling. Notably, inhibition of IKK abolished the positive effect of CT55 on NF-κB activation. Collectively, our findings strongly indicate that CT55 deficiency suppresses the development of CAC and that the CT55-TNF-α-induced NF-κB axis may represent a promising target for CAC therapy.

## Introduction

Colorectal cancer (CRC) is one of the most common malignancies with different incidences in different countries^1^. Previous research studies have proven that the pathogenesis of most CRC cases was related to environmental factors, especially intestinal symbiotic bacteria, pathogens, and chronic enteritis^2^, while only 20–30% of cases have a familial basis^3^. Colitis-associated cancer (CAC) is a CRC subtype that often shows rapid progression, with a poor response to treatment and high mortality^4^. Indeed, the development of CAC is closely related to chronic inflammation, and studies have shown that colitis patients suffering from inflammatory bowel diseases (IBDs) have an increasing risk for the development of CAC^5^. Approximately 18.4% of patients with IBD are reported to develop into CAC within 30 years after the onset of disease^2^. Thus, it is necessary to discover the targets that regulate chronic inflammation to prevent the development of CAC.

Currently, common signaling pathways, such as those involved in the Toll-like receptor signaling pathway, STAT3 signaling pathway, NF-κB signaling pathway, mitogen-activated protein kinase (MAPK) signaling pathway, Wnt signaling pathway, and epidermal growth factor receptor (EGFR) signaling pathway, were proven to have alterations in CAC^6–11^. As a key regulator of inflammation, NF-κB is likely to have a prominent role in the process of colitis-associated tumorigenesis^12,13^. More than 50% of colorectal and colitis-associated tumors and mouse studies have detected abnormal NF-κB activation^14^. Previous studies have shown that inactivation of the IKK/NF-κB pathway can attenuate the formation of inflammation-associated tumors^15^. In the canonical NF-κB pathway, NF-κB is a heterodimer and activates the transcription of target genes involved in proliferation, migration, and inflammation in response to stimulation; thus, NF-κB activation supports tumorigenesis mainly by increasing cell proliferation and angiogenesis, inhibiting cell death, and promoting cell invasion and metastasis^5,16,17^. Since NF-κB activation is closely related to CAC, identifying molecules that control NF-κB activation will provide novel targets for CAC therapy.

The CT antigen is a tumor-associated antigen that exhibits a specific expression pattern. Multiple studies have confirmed that many CT antigens have oncogenic functions and have been considered as targets for anticancer vaccines^18^. Moreover, recent findings have indicated that CT antigens are the most promising targets for tumor immunotherapy^19^. Here, we identified a novel therapy target, CT55. Previous studies have suggested that CT55 is a potential CT antigen and that CT55 is expressed in several cancers and normal testis. Specifically, the expression frequency of CT55 is 25%, 17%, 21%, and 15% in samples of liver, colon, gastric, and lung cancer tissues, respectively^20^. Another study showed that the downregulation of endogenous CT55 expression suppresses breast cancer cell growth and leads to the induction of apoptosis^21^. However, its role in CAC has not been addressed previously. In our study, we observed that CT55 is closely associated with CAC and that Ct55 deficiency alleviated inflammatory responses and decreased cell proliferation and colitis-associated tumorigenesis in a mouse AOM/DSS model. Mechanistic studies have demonstrated that CT55 interacts with IKK and exacerbates its phosphorylation, thus activating NF-κB signaling in response to TNF-α stimulation. Collectively, our data reveal a previously undiscovered function of CT55 in CAC pathogenesis and indicate that the CT55-TNF-α-induced NF-κB axis is a potential significant therapeutic target for treating CAC.

## Results

### Ct55 deficiency alleviates AOM/DSS-induced colitis-associated tumorigenesis

**Fig. 1:**
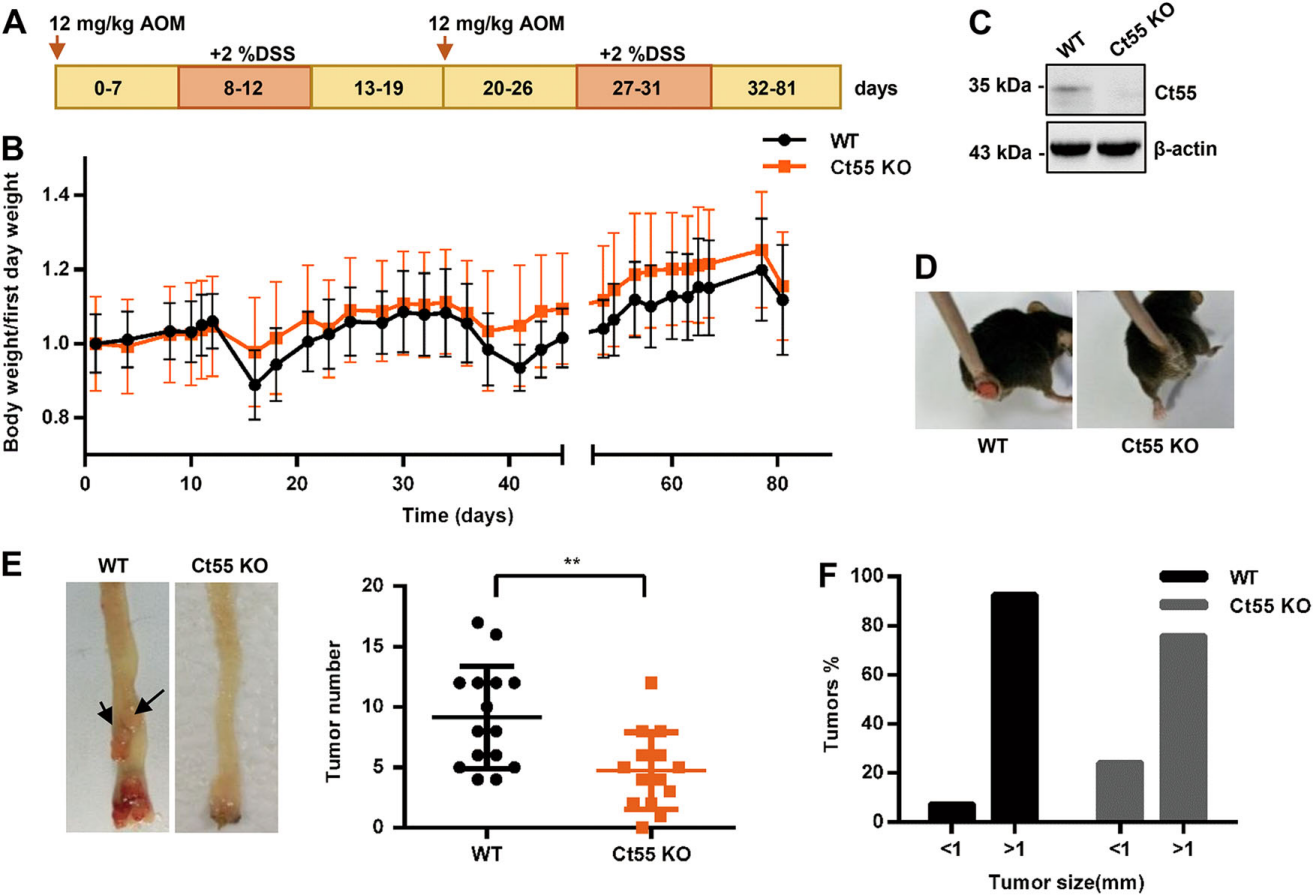
Ct55 deficiency alleviates AOM/DSS-induced colitis-associated tumorigenesis and the inflammatory response. **a** Schematic overview showing azoxymethane/dextran sulfate sodium (AOM/DSS) administration during the experiment to establish the colitis-associated colon cancer model. **b** Body weight changes during the experimental procedure in wild type (WT) and Ct55 knockout (KO) mice (*n* = 16 per group). **c** Western blot analysis of the samples from Ct55 KO and WT mice. **d** Representative images showing attenuated rectal prolapse in Ct55 KO mice compared with the WT control. **e** Tumors were measured, and the total tumor number for each animal was plotted in a line for the WT and Ct55 KO groups (*n* ≥ 14 per group). **f** Tumor size was measured, and the histogram shows the tumor size distribution (*n* ≥ 14 per group)

Several reports have indicated that CT55 is highly expressed in several cancers, including colorectal cancer^20^. To explore whether CT55 is involved in colorectal cancer, we first detected CT55 expression in 12 paired colon and adjacent nontumor human colon tissues. Interestingly, the mRNA level of CT55 was upregulated by at least two-fold in 6 out of 12 tumor tissues compared to the paired nontumor tissues (Supplementary Fig. 1a). Since CT55 has been reported to be a potential CT antigen, we next examined Ct55 expression in various tissues from wild-type (WT) mice and found that Ct55 expression was relatively higher in testis and lower in colorectum (Supplementary Fig. 1b–c). Because CT55 expression was higher in colorectal cancer than in normal colorectal tissue, it is important to investigate whether CT55 serves as a regulator in colorectal cancer. As shown in Fig. 1a, the widely used AOM/DSS model of colitis-associated colorectal tumorigenesis was applied, and WT and Ct55 knockout mice were used for the modeling. Ct55 knockout mice were generated by CRISPR/Cas9-mediated genome editing. Sanger sequencing analysis of the targeted site in samples from Ct55 knockout and control mice showed that Ct55 was absent in the Ct55 knockout mice (Supplementary Fig. 1d). Immunoblot analysis further demonstrated the loss of Ct55 protein in the colorectum of Ct55 knockout mice (Fig. 1c). During the generation of the AOM/DSS model, the body weights of mice were measured daily, and the Ct55 knockout mice exhibited less weight loss than the WT mice (Fig. 1b). Moreover, the symptom of rectal prolapse was attenuated in Ct55 knockout mice compared with the WT control (Fig. 1d), and we observed that there was a lower incidence of rectal prolapse in Ct55-deficient mice, which is 14.3% (6 out of 15) in contrast to 40% (6 out of 15) in WT mice at 60 days in our model (Supplementary Fig. 1e). In the end, the tumors were counted, and tumor size was measured. The number of macroscopically visible tumors was significantly decreased in the Ct55 knockout mice, and the tumors in the Ct55 knockout mice were markedly smaller in size compared with those in the WT mice. (Fig. 1e, f). Taken together, these observations suggest that Ct55 deficiency alleviates AOM/DSS-induced colitis-associated tumorigenesis.

### Ct55 deficiency alleviates the inflammatory response and inhibits CAC cell proliferation

**Fig. 2:**
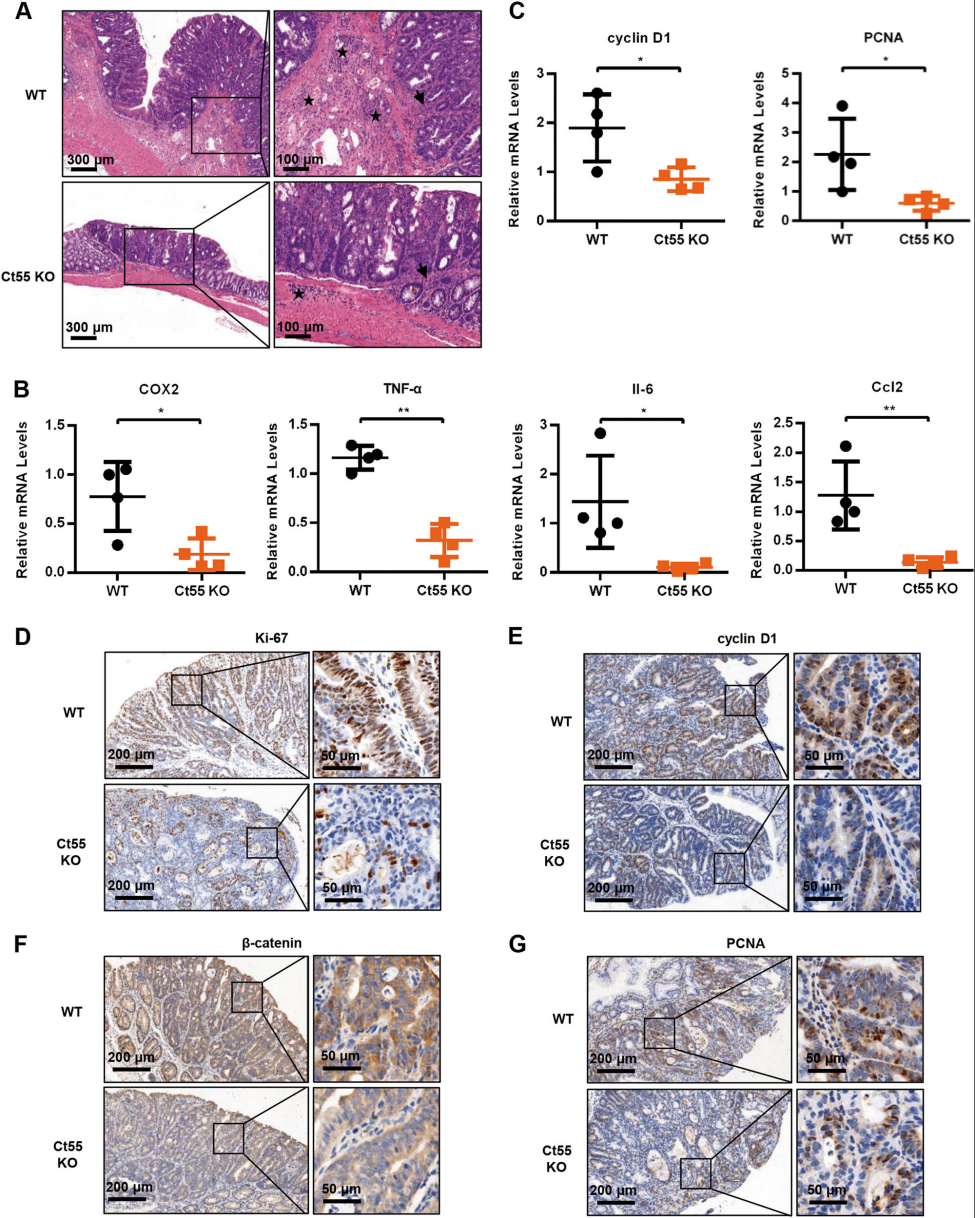
Ct55 deficiency inhibits CAC cell proliferation. **a** Representative hematoxylin and eosin (H&E) images of inflammation in the tumor tissues of Ct55 KO and WT mice after AOM/DSS treatment (*n* = 4 per group). Arrowhead: immune infiltration into the mucosa; star: immune infiltration into the submucosa. **b** Quantification of the mRNA levels of COX2, TNF-α, Il-6, and Ccl2 showed significantly lower levels in the tumor tissues of the Ct55 KO mice than in the WT mice. Cytokine expression was normalized to β-actin (*n* = 4 per group). **c** Quantitative RT-PCR further confirms that the mRNA levels of cyclin D1 and PCNA were significantly decreased in tumor tissues of the Ct55 KO mice compared with the WT mice (*n* = 4 per group). **d–g** Representative images of Ki-67, cyclin D1, β-catenin, and PCNA immunofluorescence staining in paraffin-embedded colon sections from the tumor tissues of WT and Ct55 knockout mice after AOM/DSS treatment (*n* = 4 per group)

It is widely accepted that the inflammatory response is a hallmark of colitis-associated colorectal cancer^5,22^. Therefore, hematoxylin and eosin staining was performed, and the results showed less inflammatory in Ct55 knockout mouse tumor tissues than in WT mouse tissues (Fig. 2a). Moreover, it is well known that many cytokines, such as TNF-α, COX2, and IL6, can regulate carcinogenesis by affecting cell proliferation and apoptosis; thus, the production of cytokines was examined by quantitative real-time PCR (qRT-PCR) in the tumor tissues of Ct55 knockout and WT mice. As shown in Fig. 2b, the mRNA expression levels of COX2, TNF-α, Il-6, and Ccl2 were significantly decreased in the tumor tissues of the Ct55 knockout mice compared with those of the WT mice. Decreased cytokine production may be critical for the protective effect of Ct55 deficiency in inflammation-associated carcinogenesis. In addition to the inflammatory response, increased cell proliferation has also been implicated in colitis-associated colorectal cancer. We further explored whether Ct55 regulates this process. As shown in Fig. 2c, decreased expression levels of the proliferation-related genes PCNA and cyclin D1 were observed in the tumor tissues of Ct55 knockout mice by qRT-PCR. Furthermore, the number of Ki-67-positive cells was significantly decreased in the tumor tissues of Ct55 knockout mice via Ki-67 staining (Fig. 2d). Consistently, we also found that the percentages of β-catenin-, PCNA- and cyclin D1-positive cells in the tumor tissues of Ct55 knockout mice were lower than those in the WT mice after AOM/DSS treatment (Fig. 2e–g). Altogether, our results indicate that Ct55 deficiency alleviates the inflammatory response and inhibits cell proliferation during colitis-associated colorectal cancer.

### CT55 deficiency inhibits the tumorigenic features of colorectal cancer cells

**Fig. 3:**
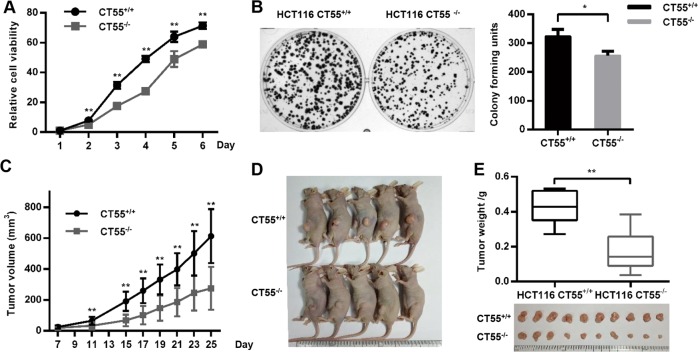
CT55 deficiency inhibits the tumorigenic features of colorectal cancer cells. **a** CCK8 assays were performed to evaluate the cell viability of HCT116 CT55 KO cells compared with the WT control (*n* = 6, **P < 0.01). **b** Relative colony formation units of HCT116 WT and HCT116 CT55 KO cells (left). The quantification of the indicated relative colony-forming units (*n* = 3, *P < 0.05) (right). **c** The tumor volumes of the HCT116 and HCT116 CT55 KO-treated mice 25 days after transplantation. **d** Representative images of the injected mice (*n* = 5, **P < 0.01). **e** Tumor weights and images of the isolated tumors from the injected mice (*n* = 10, **P < 0.01)

Since Ct55 deficiency alleviates AOM/DSS-induced colitis-associated tumorigenesis, we then assessed the role of CT55 in the growth of colorectal cancer cells, and HCT116 CT55 KO cell lines were prepared using CRISPR/Cas9-mediated genome editing. As shown in Supplementary Fig. 2a and Fig. 5c, knockout was detected by Sanger sequencing and western blot analyses. Then, cell viability was determined using Cell Counting Kit-8 (CCK8) assays, and we observed that CT55 deficiency decreased the cell viability of HCT116 cells (Fig. 3a). To examine the effect of CT55 upon proliferation, a colony formation assay was performed, and we observed a significant decrease in the number of colony-forming units by HCT116 CT55 KO cells compared with HCT116 WT cells (Fig. 3b). Moreover, a BALB/c nude mouse xenograft model was applied to evaluate the effect of CT55 on tumorigenicity. As shown in Fig. 3c, the tumor volume was reduced with CT55 deficiency. Also, the weight of CT55-deficient xenograft tumors at sacrifice was smaller in contrast to control (Fig. 3d, e), suggesting that CT55 deficiency effectively suppressed tumor growth in nude mice. These results demonstrate that CT55 deficiency inhibits the tumorigenic features of colorectal cancer cells.

### Function of CT55 in CAC depends on NF-κB signaling

**Fig. 4:**
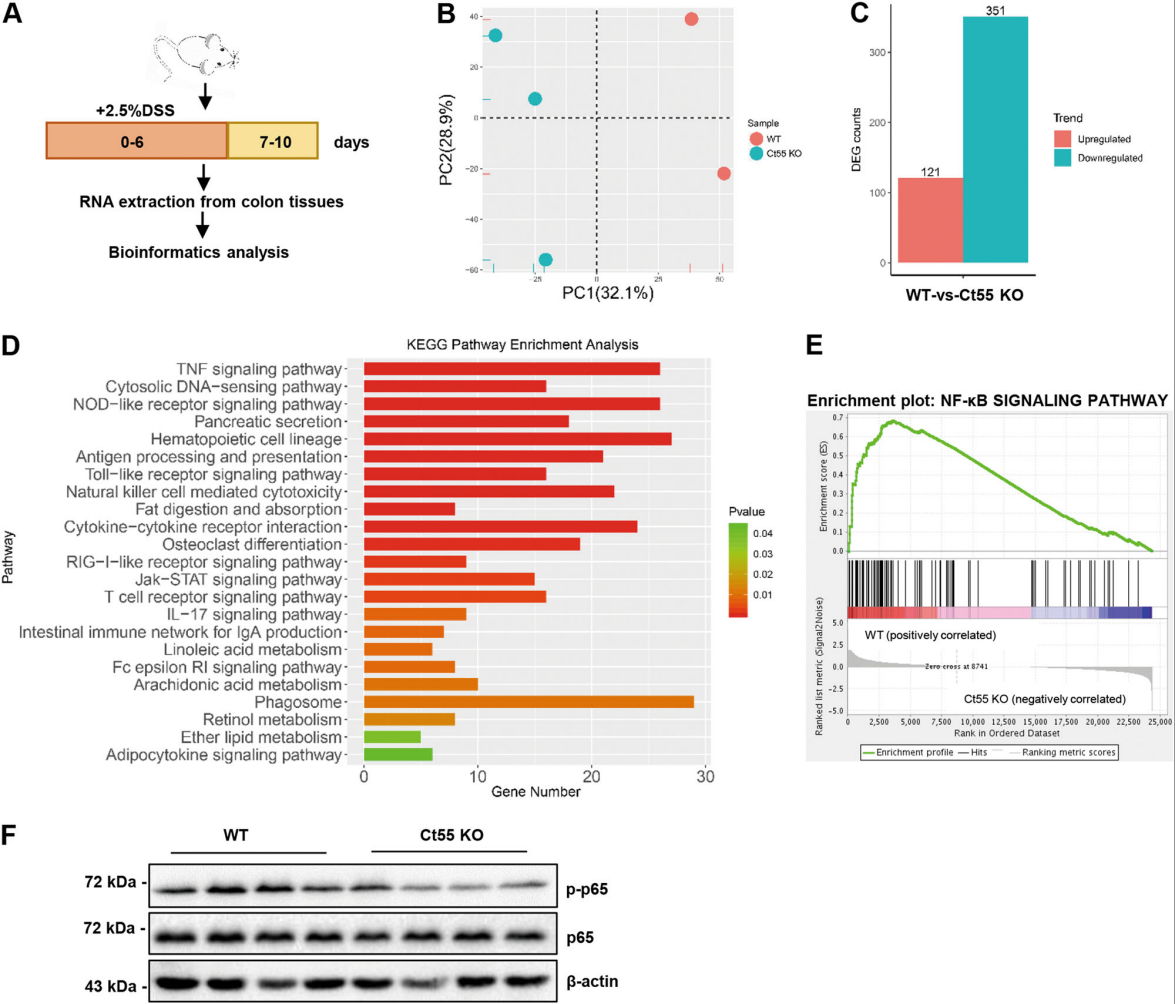
The function of CT55 in CAC depends on NF-κB signaling. **a** Schematic of the DSS-induced colitis used to identify the relevant pathway of Ct55 function in colitis-associated cancer (CAC). **b** Principal component analysis shows two distinct clusters for the WT and Ct55 KO samples (WT *n* = 2, Ct55 KO *n* = 3). **c** A column diagram shows the differentially expressed genes between WT and Ct55 KO groups. Gene sets with a fold change >2 and false discovery rate (FDR) < 0.05 were considered statistically significant. **d** Kyoto Encyclopedia of Genes and Genomes (KEGG) pathway enrichment analysis of the most significantly enriched pathways (*P* < 0.05 by Fisher’s exact test) on the basis of the identified differentially expressed genes between the WT and Ct55 KO groups. **e** Gene set enrichment analysis (GSEA) of the NF-κB signaling pathway in the WT and Ct55 KO groups. **f** Western blot analysis of the indicated protein levels in tumor tissues of the WT and Ct55 KO mice. β-Actin served as the loading control

Ct55 deficiency is known to alleviate AOM/DSS-induced colitis-associated tumorigenesis. We aimed to gain further insight into the full spectrum of Ct55 functions in AOM/DSS-induced colitis-associated tumorigenesis at the molecular level. Given the finding that Ct55 significantly decreased the inflammatory response in the AOM/DSS model and its reported role in immunotherapy, we performed RNA-seq analysis of the mouse colon samples obtained from Ct55 knockout and WT mice that underwent DSS colitis experiments (Fig. 4a). First, principal component analysis was performed with the mouse colon tissue data, and we found that the samples from the Ct55 knockout and WT mice were clearly separated (Fig. 4b). Then, Kyoto Encyclopedia of Genes and Genomes (KEGG) pathway enrichment analysis was carried out to identify the most significantly enriched pathways. Among the differentially expressed genes, 121 genes were upregulated and 351 genes were downregulated in Ct55 knockout samples compared with WT samples (Fig. 4c). On the basis of the number of changed genes and their statistical significance, a large number of pathways associated with immune and inflammatory responses were observed in the case of Ct55 knockout. In terms of RNA expression levels, the immune and inflammatory responses were inhibited in the Ct55 knockout mice compared with the WT mice (Supplementary Fig. 3a), which was consistent with previous data from the AOM/DSS mouse model. Notably, the TNF signaling pathway was affected the most by Ct55 knockout, as indicated by the number of genes altered at a statistically significant level (Fig. 4d). It is well recognized that the activation of the TNF pathway leads to the activation of NF-κB signaling^23,24^, and many studies have shown that NF-κB signaling plays a crucial role in colitis-associated colorectal cancer^25^. Thus, gene set enrichment analysis was carried out to detect the enrichment of the NF-κB signaling pathway between the WT and Ct55 knockout groups. As expected, the NF-κB signaling pathway was enriched in the WT group but not in the Ct55 knockout group (Fig. 4e). To verify the hypothesis that Ct55 may function in colitis-associated colorectal cancer by targeting TNF-α-induced NF-κB signaling, we detected the expression levels of p-p65 and other indicated proteins by western blot. As expected, colon tissues from the Ct55 knockout mice showed decreased phosphorylation levels of p65 compared with those from the WT mice (Fig. 4f). Collectively, these data suggest that the Ct55 functions in CAC are dependent on TNF-α-induced NF-κB signaling.

### CT55 is a positive regulator of TNF-α-induced NF-κB signaling

**Fig. 5:**
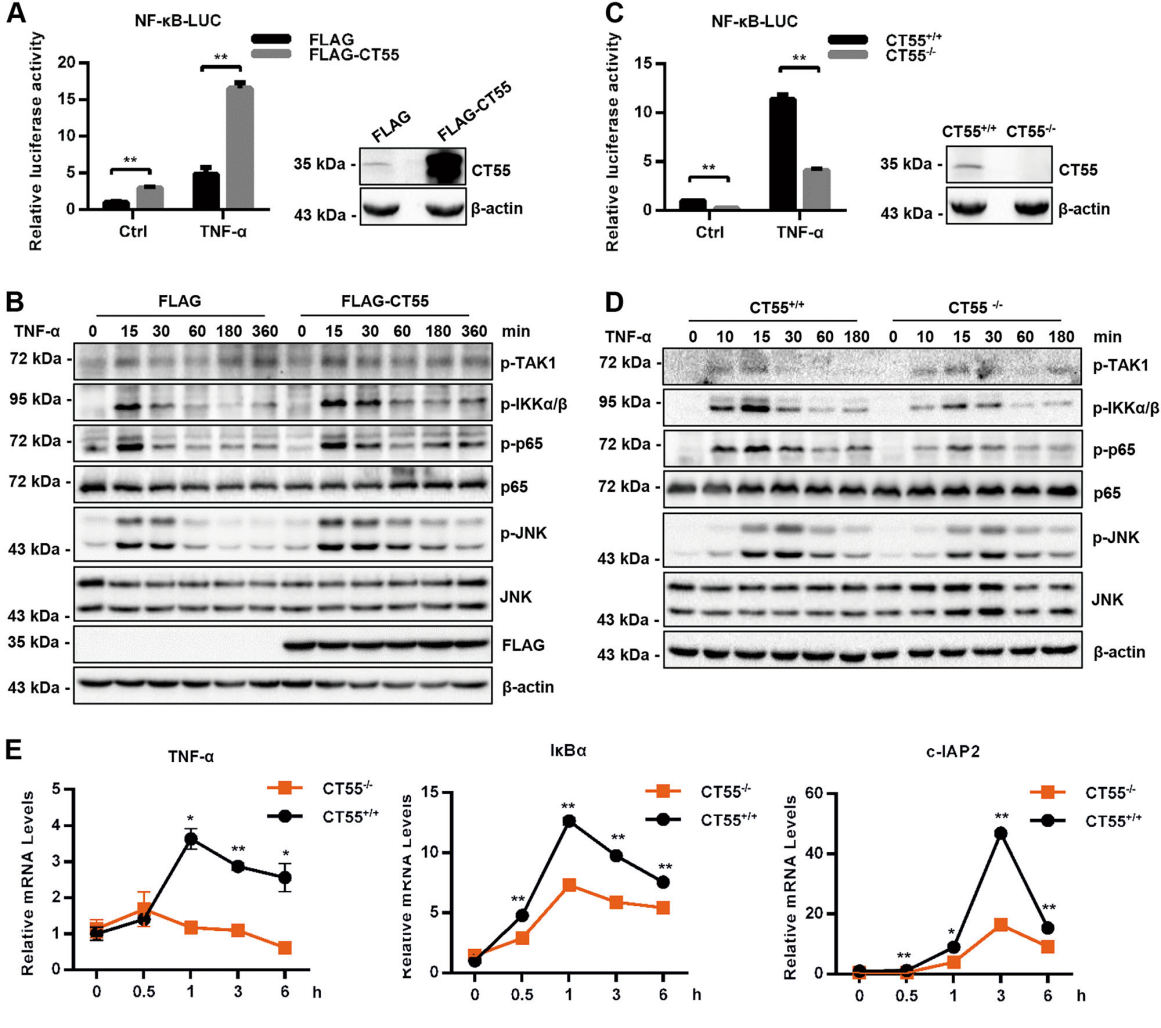
CT55 is a positive regulator of TNF-α-induced NF-κB signaling. **a** Luciferase experiments were performed to evaluate TNF-α-induced NF-κB activation in HCT116 cells with or without CT55 overexpression (left). Immunoblot analysis of HCT116 cells transfected with plasmids encoding FLAG-tagged CT55 and FLAG control (right). **b** Western blot analysis of the indicated protein levels in HCT116 cells transfected with FLAG-CT55 for 24 h and treated with TNF-α (20 ng/ml) for the indicated times. β-Actin served as the loading control. **c** TNF-α-induced NF-κB luciferase activity in HCT116 cells with or without CT55 (left). Immunoblot analysis of the knockout efficiency (right). **d** Protein expression levels of the indicated proteins from the HCT116 cells with or without CT55 that were treated with TNF-α (20 ng/ml) for the indicated times were analyzed by western blotting. β-Actin served as the loading control. **e** The mRNA level associated with the TNF-α-induced transcription of the endogenous TNF-α, IκBα, and c-IAP2 genes in HCT116 cells with or without CT55

As noted above, RNA-seq analysis and western blotting showed that Ct55 deficiency played a protective role in colitis-associated colorectal cancer by regulating TNF-α-induced NF-κB signaling. To obtain more evidence supporting the role of CT55 in TNF-α-induced NF-κB activation, in vitro experiments were carried out in human colon cancer cells. Overexpression and knockout efficiency are shown in Fig. 5a, c. As shown in Fig. 5a, the luciferase reporter assay showed that NF-κB activity was higher in the CT55-overexpressing cells than in the control cells, especially in the condition of TNF-α stimulation. In TNF-α-induced NF-κB activation, stimulation of the TNF receptor (TNFR) leads to activation of the TAK1 complex through TRAF proteins. TAK1 then activates IKK, which phosphorylates IκB proteins. Ubiquitinated IκB is degraded, leading to translocation of the p50/p65 NF-κB dimer from the cytoplasm to the nucleus and activation of gene transcription. Thus, we further studied whether CT55 could affect the phosphorylation of TAK1, IKK, and p65 upon stimulation with TNF-α. Consistent with the results of the luciferase reporter assay, we observed by western blot that the activation of p65 and its upstream kinases IKK was potentiated in the CT55-overexpressing HCT116 cells compared with the control, while the activation of TAK1 resulted in almost no change. It was also obvious that TNF-α-induced JNK activation was markedly increased in the CT55-overexpressing cells compared with the control (Fig. 5b). In contrast, marked reductions in NF-κB luciferase activity and p65 signaling were observed in the CT55 knockout HCT116 cells compared with the parental HCT116 cells (Fig. 5c, d). Furthermore, qRT-PCR analyses indicated that knockout of CT55 inhibited transcription of TNF-α-induced NF-κB downstream genes, including TNF-α, IκBα, and c-IAP2, at the indicated time points, while overexpression of CT55 had no significant effects (Fig. 5e). In accordance with the data obtained in vivo, these data suggested that CT55 positively regulated TNF-α-induced NF-κB signaling cascades.

### CT55 regulates NF-κB activity by interacting with the IKK complex

**Fig. 6:**
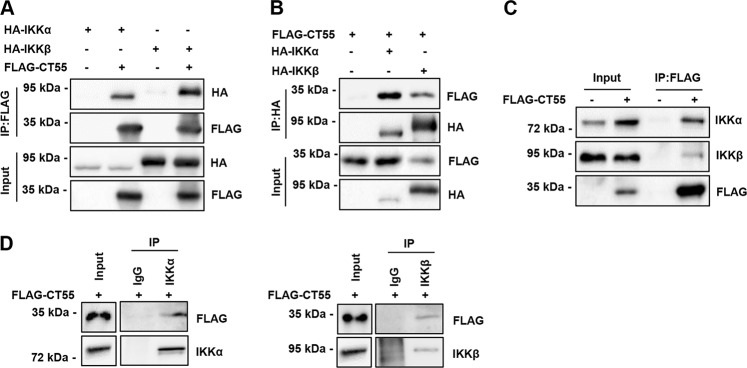
CT55 regulates NF-κB activity by interacting with the IKK complex. **a, b** Immunoprecipitation and immunoblot analysis of HCT116 cells transfected with FLAG-tagged CT55 and HA-tagged IKKα and IKKβ for 24 h and treated with TNF-α (20 ng/ml) for 6 h. The lysates were immunoprecipitated with anti-Flag or anti-HA antibody. Then, western blotting was performed to detect the indicated proteins. **c** Western blot analysis of overexpressed CT55 after immunoprecipitation of IKKα and IKKβ in HCT116 cells treated with TNF-α (20 ng/ml) for 6 h. **d** Western blot analysis of IKKα and IKKβ after immunoprecipitation of FLAG-CT55 in HCT116 cells treated with TNF-α (20 ng/ml) for 6 h

To further elucidate the positive role of CT55 in TNF-α-induced activation of NF-κB signaling, we next performed immunoprecipitation to evaluate potential interactions between CT55 and components of the NF-κB pathway. TAK1 and IKK are two pivotal signaling molecule leading to the activation of the transcription factors NF-κB. Because CT55 can affect the phosphorylation of the core protein p65 and the upstream IKK protein complex but not the TAK1, coimmunoprecipitation was performed in HCT116 cells cotransfected with HA-tagged IKK and FLAG-tagged CT55. Not surprisingly, the results showed that CT55 interacted with the IKK complex (Fig. 6a, b). To further confirm the interaction, another coimmunoprecipitation experiment was performed in HCT116 cells transfected with FLAG-tagged CT55 only, and the results showed that endogenous IKK interacted with FLAG-tagged CT55 (Fig. 6c, d). These data suggest that CT55 functions in TNF-α-induced NF- κB signaling by interacting with the IKK complex.

### CT55 regulates IκBα degradation and p65 nuclear localization mainly in an IKK-dependent manner

**Fig. 7:**
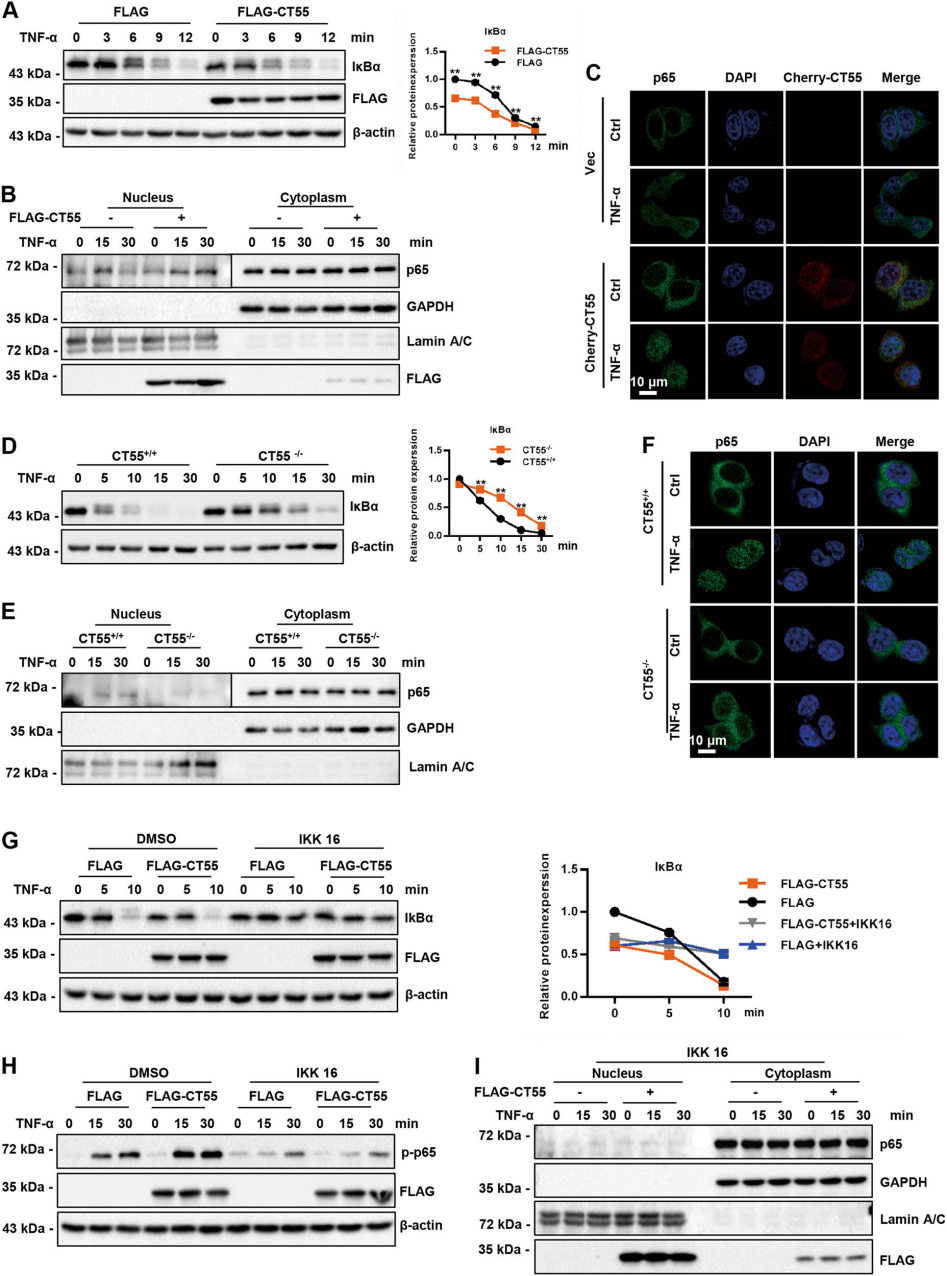
CT55 regulates IκBα degradation and p65 nuclear localization and mainly in an IKK-dependent manner. **a** Overexpression of CT55-accelerated IκBα degradation following TNF-α treatment. **b**, **c** Overexpression of CT55 potentiated p65 translocation to the nucleus following TNF-α treatment. **d** Deficiency of CT55 inhibited IκBα degradation following TNF-α treatment. **e**, **f** Deficiency of CT55 inhibited p65 translocation to the nucleus following TNF-α treatment. For **a** and **d**, HCT116 cells transfected with Flag-CT55 (**a**) or KO CT55 (**d**) were treated with TNF-α (20 ng/ml) at the indicated times. Western blotting was performed to detect the protein level of IκBα. The normalized figure is presented at the right. β-Actin was used as a loading control. For **b** and **e**, HCT116 cells transfected with Flag-CT55 (**b**) or cells with CT55 KO (**d**) were treated with TNF-α (20 ng/ml) at the indicated times and then separated into cytoplasmic and nuclear fractions. Western blot analysis was performed using the p65 antibody. Lamin A/C was used as the nuclear loading control, and GAPDH served as the cytoplasmic loading control. For **c**, HeLa cells transfected with Cherry-CT55 were treated with or without TNF-α (20 ng/ml) for 15 min. Then, immunofluorescence staining was carried out. For **f**, CT55 KO HCT116 cells were treated with or without TNF-α (20 ng/ml) for 30 min. Then, immunofluorescence staining was performed. **g–i** IKK 16 treatment overwhelmed the function of CT55 to promote the activation of p65 and IκBα degradation. HCT116 cells transfected with Flag-CT55 were treated with IKK 16 (20 mM) or DMSO for 2 h before TNF-α treatment. Western blotting was performed to detect the protein levels of IκBα, p-p65, and p65

NF-κB controls the expression of various genes involved in the process of inflammatory, antiapoptotic, and immune responses. Overwhelming evidence has shown that the activation of NF-κB is controlled through its cytoplasmic sequestration by IκB kinase. In response to TNF-α stimuli, IκB kinase is phosphorylated by its upstream kinase, the IKK complex, and is targeted for ubiquitination- and proteosome-dependent degradation. Then, NF-κB is released and translocated into the nucleus, triggering the transcription of downstream genes^26^. Therefore, IκBα degradation and nuclear translocation of p65 are two pivotal steps in NF-κB activation. As shown in Fig. 7a, after treatment with TNF-α for the indicated time, the protein level of IκBα in CT55-overexpressing HCT116 cells decreased significantly. Moreover, overexpressed CT55 facilitated p65 translocation into the nucleus upon stimulation with TNF-α (Fig. 7b). Consistently, the immunofluorescence staining analysis displayed similar results (Fig. 7c). In contrast with CT55 overexpression, the CT55 knockout HCT116 cells exhibited markedly higher IκBα protein levels and less p65 nuclear localization than the WT controls in response to TNF-α stimulation (Fig. 7d, f). These results suggest that CT55 may affect TNF-α-induced NF-κB signaling by accelerating the degradation of IκBα and increasing nuclear p65.

To ascertain the critical role of IKK in CT55-mediated NF-κB activation in vitro, we further used a specific IKK inhibitor, IKK 16, to block the activity of the IKK complex before TNF-α treatment. As shown in Fig. 7g, the protein level of IκBα in CT55-overexpressing HCT116 cells showed no obvious change after treatment with IKK 16. Furthermore, IKK 16 treatment overwhelmed the function of CT55, promoting the phosphorylation of p65 (Fig. 7h). Consistently, IKK 16 treatment potently abrogated the positive effect of CT55 on p65 nuclear localization (Fig. 7i). These results indicate that IKK is required for CT55-mediated NF-κB activation.

## Discussion

Aberrant NF-κB activation is a significant cause of CAC progression. It has been reported that cancer cells with NF-κB pathway activation were resistant to chemotherapeutics and ionizing radiation, while inhibition of NF-κB activity effectively increased the cell sensitivities to these agents^25^. These findings indicated that NF-κB has the potential to improve the efficacy of cancer therapies. Surprisingly, we found that Ct55-deficient mice were resistant to colitis-associated tumorigenesis in the animal AOM/DSS model by targeting TNF-α-induced NF-κB signaling. In previous studies, CT55 was described as a potential novel CT antigen that has been detected in several cancers, such as lung, gastric, and cervical cancers, but not in normal tissues^20^. In our study, we found that CT55 expression was elevated in human colorectal cancer, whereas it was low in normal mouse tissues, except for the testis (Supplementary Fig. 1a–c). These results indicated that CT55 may be involved in colitis-associated colon tumorigenesis. Additionally, CT55 has been reported to enhance the growth of breast cancer cells by binding to the BRCA1 gene^21^. Similarly, we are the first to report that CT55 is a strict regulator of CAC, and we extend our research using a well-accepted animal AOM/DSS model. The model was established with injection of AOM followed by repeated oral administration of DSS in the drinking water, which causes chronic inflammation in the colon and ultimately promotes CAC formation. Notably, compared with the WT mice, the mice with Ct55 deficiency exhibited decreased colonic inflammation and cell proliferation. Consistently, the tumor incidence and size were also decreased in the Ct55-deficient mice. In the HCT116 cell model, we found that CT55 deficiency decreased cell viability and inhibited cell proliferation and the tumorigenicity of HCT116 cells. These data indicate that CT55 could be a novel target for CAC treatment.

A recent study reported that CT55 is a potential novel immune target for achieving treatment-free remission in chronic myeloid leukemia patients^27^, which prompted us to investigate whether CT55 affects immune and inflammatory responses during the process of CAC. Analysis of the mechanisms by which CT55 mediates a significant effect on CAC revealed that TNF-α-induced NF-κB signaling is the major downstream target. Chronic inflammation is an important risk factor in promoting tumorigenesis. As a key regulator of inflammation, NF-κB can be activated to stimulate the expression of proinflammatory cytokines, antiapoptotic genes, angiogenesis factors, and proteases to promote tumor initiation, survival, and proliferation as well as the invasion of malignant cells in the development and progression of CAC^25^. Consistent with previous studies, Ct55-deficient mice showed decreases in the incidence and size of colitis-associated tumors as a consequence of decreased NF-κB activity. Our mechanistic study found that CT55 could interact with the IKK complex, increasing its phosphorylation activity and accelerating IκBα degradation and p65 nuclear localization in HCT116 cells. Inhibition of IKK abolished the positive effect of CT55 on NF-κB activation. Nucleus-located p65 was reported to regulate the transcription of numerous genes, including TNF-α. Adverse effects of TNF-α in tumor initiation and progression have also been suggested, and there has been some evidence that treating with the TNF-α antagonist etanercept reduced mucosal inflammatory cell infiltration, tumor incidence, and tumor size^23^. Accordingly, combined with the results of RNA-seq in mouse colitis, our study showed that CT55 could regulate the production of TNF-α by affecting NF-κB activation in HCT116 cells, which might explain how CT55 functions in CAC.

Notably, recent studies have indicated that the effect of the NF-κB pathway on tumor growth in either suppressing apoptosis or inducing proinflammatory cytokines depends on different cell types. In enterocytes, conditional knockout of IKKβ can reduce tumor incidence by 80% without affecting the size of the tumor, indicating that NF-κB functions in early stages of tumor promotion but not in progression or growth. In contrast, deletion of IKKβ in inflammatory cells decreased tumor incidence by just 50% but resulted in a marked reduction in tumor size, suggesting that NF-κB functions mainly in progression or growth^25^. In our experiments, not only the tumor incidence but also the tumor size were decreased in Ct55-deficient mice. Mechanistically, we explored the effect of CT55 on NF-κB activation in colon cancer cells only. Further experiments are needed to determine whether Ct55 regulates tumor size by affecting NF-κB activation in inflammatory cells. Moreover, marked changes in TNF-α-induced JNK activation were detected in CT55-overexpressing and CT55 knockout cells; thus, it is necessary to explore whether CT55 contributes to CAC partly by regulating TNF-α-induced JNK activation.

In summary, our study revealed the crucial involvement of CT55 in regulating the incidence and development of CAC by controlling the TNF-α-induced NF-κB signaling pathway. Specifically, we found that Ct55-deficient mice were resistant to CAC in a colitis-associated colon cancer model. Mechanistic studies showed that CT55 interacts with the IKK complex and increases the phosphorylation of IKK, thereby activating IKK–p65 signaling, while knockout of CT55 blocks IKK–p65 signaling. These findings provide a novel therapeutic target to significantly reduce the risk of CAC.

## Materials and methods

### Tissue specimens

CRC and adjacent nontumor colon tissues were collected from patients undergoing CRC resection. Tissue samples were immediately frozen in liquid nitrogen and stored. Informed consent was obtained at the Tongji Medical College Huazhong University of Science & Technology in Wuhan, China. None of the patients received chemotherapy prior to colectomy. All patients were unrelated ethnic Han Chinese. Informed consent was obtained from all subjects.

### Generation of genetically modified mice

Ct55 knockout mice were obtained by clustered regularly interspaced short palindromic repeats (CRISPR)/Cas9-mediated genome editing^28^. Briefly, a single guide RNA (sgRNA) targeting Ct55 intron 1 was designed. First, the vectors encoding Cas9 (44758; Addgene) and guide RNA (gRNA) were transcribed in vitro into messenger RNA (mRNA) and gRNA. Second, the transcribed mRNA and gRNA were injected into fertilized eggs that were transplanted into pseudopregnant mice. Next, genome DNA fragments from F0 mice were amplified by PCR, and the selected chimeras were crossed with WT C57BL/6 mice. Then, the F1 Ct55^−/+^ mice were crossed with WT C57BL/6 mice for at least three generations. The mice were genotyped by PCR analysis followed by sequencing, and the resulting Ct55^−/+^ mice were crossed to generate Ct55^−/−^ and Ct55^+/+^ mice. Ct55^−/−^ and Ct55^+/+^ littermates were cohoused until weaning age and then separated for experiments. The sequences of the sgRNA and Ct55 check primers are shown in Supplementary Table 1

### Animals and treatment

All mice (8–10 weeks old) were housed in a specific pathogen-free and temperature-controlled (23 ± 2 °C) environment with a 12-h light/dark cycle. All animal experiments were conducted in accordance with protocols approved by the Institutional Animal Care and Use Committee of Wuhan University.

The mouse AOM/DSS model was generated as described previously^29^. Briefly, mice were first injected intraperitoneally with 12 mg/kg AOM (Sigma-Aldrich, St Louis, MN) dissolved in phosphate-buffered saline (PBS). Seven days after injection, 2% DSS (35–50 kDa; MP Biochemicals, Solon, OH) was added to their drinking water for five consecutive days followed by 7 days of regular drinking water. Then, the mice received a second intervention of AOM (12 mg/kg). On the seventh day after the second AOM intervention, 2% DSS was added again to the drinking water for five consecutive days followed by regular water until day 81. During the modeling process, the body weight, diarrhea, and hematochezia were monitored. Mice were sacrificed on day 81.

For the mouse DSS-induced colitis model, water with 2.5% DSS was given to the mice for 6 days and was then replaced with regular water for 4 days^30^. The mice were killed after the study.

### Histology and immunohistochemistry staining

Colon segments were fixed in 4% paraformaldehyde solution, dehydrated, paraffin-embedded, and sectioned into 5-μm sections. Hematoxylin and eosin (H&E) and immunohistochemistry staining was performed as previously reported^31^. Histopathology images were captured with a light microscope (Olympus, Tokyo, Japan).

### Generation of the CT55 knockout cell line

CT55 knockout in HCT116 cells was obtained by clustered regularly interspaced short palindromic repeats (CRISPR)/Cas9-mediated genome editing. The sgRNA and CT55 check primers used are shown in Supplementary Table 1.

### Cell viability and colony formation assay

For CCK8 assays, cells were seeded at a density of 1 × 10^3^ cells/well in 96-well plates. On days 1–6, 10 μl of CCK8 solution (Dojindo Laboratories, Kumamoto, Japan) was diluted to 100 μl and was added to each well of the plate and then incubated for 1 h at 37 °C. Then, cell viability was determined by using a spectrophotometer (ELx800, BioTek, USA) set at a wavelength of 450 nm. For colony formation assays, HCT116 WT and HCT116 CT55 KO cells were seeded in a six-well plate at 500 cells/well and grown for 14 days. Then, clones were stained with crystal violet and photographed. Each experiment was repeated three times.

### Tumorigenicity assays in nude mice

All experimental procedures involving animals were performed in accordance with the Guide for the Care and Use of Laboratory Animals and were approved by the Animal Care and Use Committee of Wuhan University. A total of 5 × 10^6^ cells were suspended in 200 μl of PBS and injected subcutaneously into the flanks of female athymic nude mice. Five mice were injected per group. Tumor volume was measured every two days. The tumor volume (*V*) was monitored by measuring the length (*L*) and width (*W*) of the tumor with calipers and was calculated using the formula *V* = (*L* × *W*^2^) × 0.5.

### RNA extraction and quantitative real-time PCR

Total RNA was extracted from frozen colon tissues and cells using TRIzol reagent (#15596-026; Invitrogen, Carlsbad, CA, USA), and the RNA was reverse-transcribed into cDNA by the Transcriptor First-Strand cDNA Synthesis Kit (#04896866001; Roche, Munich, Germany) according to the manufacturer’s instructions. The mRNA level of each gene was normalized to β-actin. The primers used to amplify specific gene fragments are shown in Supplementary Table 2.

### Western blot analysis

Proteins (30 μg/sample) from cell cultures or colon tissue samples were separated via sodium dodecyl sulfate polyacrylamide gel electrophoresis gel and were transferred to PVDF membranes (IPVH00010; Millipore, Billerica, MA, USA). Then, the membranes were blocked with 5% nonfat milk in TBS and probed with primary antibodies overnight at 4 °C, followed by incubation with an HRP-conjugated secondary antibody. Finally, a ChemiDoc™ MP Imaging System (Bio-Rad, Hercules, CA, USA) was used for signal detection.

### KEGG pathway and gene set enrichment analysis

The annotation information of genes involved in biological pathways was obtained from the Kyoto Encyclopedia of Genes and Genomes (KEGG) database. Our study used clusterProfiler as an enrichment analysis tool. Gene sets with a fold change>2 and false discovery rate (FDR)<0.05 were considered statistically significant.

### Luciferase reporter assay

Cells were seeded in 24-well plates at a density of 2 × 10^5^ cells/well and transfected with the indicated plasmids for 24 h. Luciferase assays were performed using a dual-luciferase assay kit (Promega). NF-κB luciferase activity was normalized to RI-TK luciferase activity.

### Cell culture

The human colorectal cancer cell lines HCT116 (from ATCC) and CT55 KO HCT116 were cultured in McCoy’s 5A medium (AppliChem, Darmstadt, Germany) supplemented with 10% fetal bovine serum (FBS; HyClone, Logan, UT, USA) and 100 U of penicillin–streptomycin (Gibco, Carlsbad, CA, USA). HEK293T cells (from ATCC) and HeLa cells (from ATCC) were grown in complete DMEM (HyClone) supplemented with 10% FBS and 100 U of penicillin–streptomycin. All cells were cultured at 37 °C in a 5% CO_2_ incubator.

### Plasmid constructs

All plasmids were constructed following standard molecular biology techniques. The sequences of human CT55 were amplified via PCR, and the amplified fragment was inserted into a modified pcDNA5 expression vector that was a gift from Prof. Min Wu (College of Life Sciences, Wuhan University). The NF-κB luciferase reporter plasmid and mammalian expression plasmid IKKα/β were previously described^32^. CT55 knockout plasmids were constructed by inserting an sgRNA into the vectors (51133; Addgene) and another plasmid vector encoding Cas9 (pST1374-NLS-flag-linker-Cas9, 44758; Addgene). The sequences of the sgRNA and CT55 screening primers are shown in Supplementary Table 1

### Coimmunoprecipitation

Cells were collected and lysed in lysis buffer (20 mM Tris-HCl, pH 7.4, 150 mM NaCl, 1 mM EDTA) containing Complete Protease Inhibitor (04693132001; Roche). Then, the lysates were centrifuged at 12,000*g* for 15 min at 4 °C. Some of the supernatant was removed as total lysate, while the rest was collected and incubated with the indicated antibodies and protein G-agarose beads (#11719416001; Roche) for 4 h at 4 °C. The immunoprecipitates were washed three times and evaluated via western blotting.

### Immunofluorescence

The cells were fixed with 4% paraformaldehyde and permeabilized with 0.1% Triton X-100 in PBS. Then, the cells were washed with PBS and blocked with 5% bovine serum albumin in PBS for 30 min and incubated with primary antibody overnight at 4 °C. Samples were washed three times and incubated with secondary antibody for 30 min. Cells were then stained with DAPI. The coverslips were mounted onto glass slides with anti-fade solution. Finally, the slips were observed and digitally photographed using a confocal microscope.

### Antibodies and reagents

HA (#M180-3) and Flag (#M185-3L) antibodies were procured from MBL Beijing Biotech Co.; β-actin (#ab8226) antibody was obtained from Abcam (Cambridge, MA, USA); GAPDH (cat# CW0266A) antibody was purchased from Beijing Cowin Biotech Co., Ltd (Beijing, China); and IκBα (#4814), p65 (#4764), p-p65 (#3033), p-IKKα/β (#2697), lamina (#4777S), PCNA (#2586), cyclin D1 (#2978), β-catenin (#9582), and Ki-67 (#9449) antibodies were obtained from CST Inc. (Beverly, Massachusetts, USA). CT55 antibodies were obtained from Sigma-Aldrich (SAB1407601). The secondary antibodies were peroxidase AffiniPure goat anti-rabbit-IgG (H+L) (#111-035-003) and goat anti-mouse-IgG (H+L) (#115-035-003) and were obtained from Jackson ImmunoResearch (Newmarket, UK). Anti-Flag M2 was purchased from Sigma-Aldrich Co. LLC (St. Louis, MO, USA). Recombinant human TNF-α (R&D systems) and luciferase reporters (Qiagen) were purchased from the indicated manufacturers. IKK 16 was purchased from MCE (Shanghai, China).

### Statistical analysis

All values in this study are expressed as the means ± SDs, and the analysis was performed using Prism 5.0 (GraphPad Software, San Diego, CA). Two-tailed Student’s *t*-test was used to compare two groups when the data showed a normal distribution and homogeneity of variance. Statistical significance was set based on the *P* value. n.s., *P* > 0.05; *, *P* < 0.05; **, *P* < 0.01.

## Supplementary information

Supplementary figure 1

Supplementary figure 2

Supplementary figure 3

Supplementary figure 4

Supplementary Table
