# Supplementary figures and images for: Cancer testis antigen 55 deficiency attenuates colitis-associated colorectal cancer by inhibiting NF-κB signaling
Source: Cell Death Dis. 2019 Apr 3;10(4):304. doi: 10.1038/s41419-019-1537-x (PMC6447546; doi:10.1038/s41419-019-1537-x)

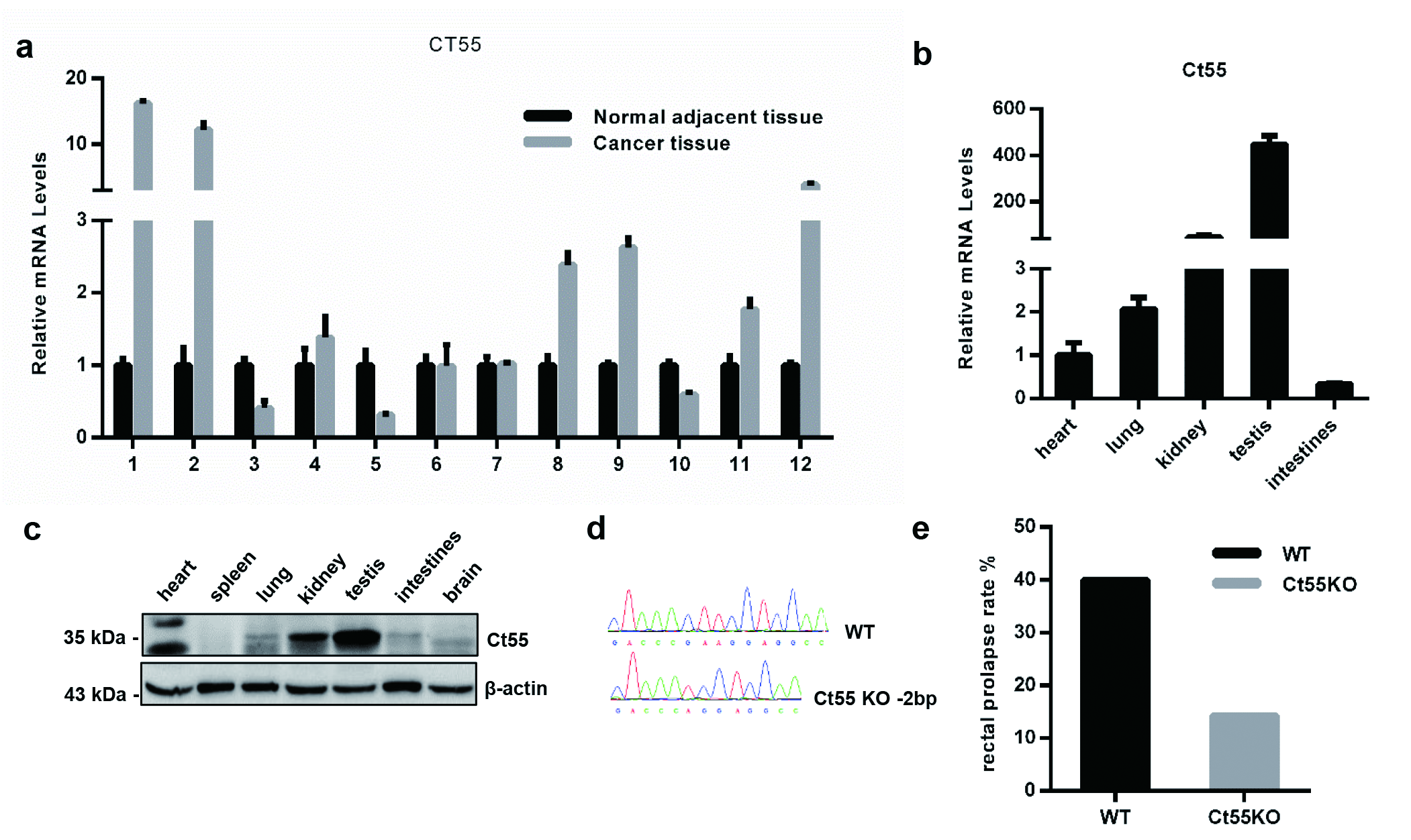

Supplement: Supplementary file 1 — Supplementary figure 1 [file 41419_2019_1537_MOESM1_ESM.tif]

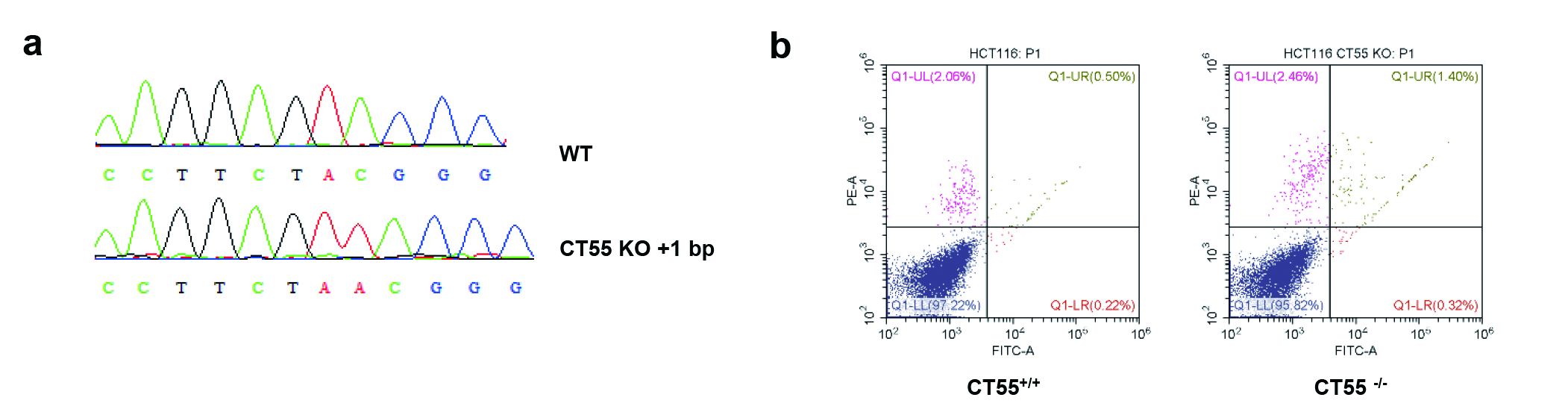

Supplement: Supplementary file 2 — Supplementary figure 2 [file 41419_2019_1537_MOESM2_ESM.tif]
