# Supplementary material for: Cancer testis antigen 55 deficiency attenuates colitis-associated colorectal cancer by inhibiting NF-κB signaling
Source: Cell Death Dis. 2019 Apr 3;10(4):304. doi: 10.1038/s41419-019-1537-x (PMC6447546; doi:10.1038/s41419-019-1537-x)
Supplement: Supplementary file 5 — Supplementary Table [file 41419_2019_1537_MOESM5_ESM.docx]

**Supplementary data:**

**Cancer testis antigen 55 deficiency attenuates** **colitis-associated colorectal cancer by inhibiting NF-κB signaling**

Huan Zhao^1^, Wen-Ming Pan^2^, Hui-Hui Zhang^4^, Yang Song^1^, Jie Chen^1^, Ying Xiang^1^, Bo Gu^1^, Shang-Ze Li^3^, Run-Lei Du ^1^, Xiao-Dong Zhang^1^

**Supplementary Tables**

**Supplementary Table 1: Sequences of the sgRNA and primers for genotyping.**

| Primer | Sequence 5'---3' |
| --- | --- |
| Mouse Ct55 sgRNA | AAGGAAAGCAGACCCGAAGGAGG |
| Mouse Ct55 check F1 | CGTAGAATTGGCGTCTGTGA |
| Mouse Ct55 check R1 | GGAGACAAAATGGTGCTGCT |
| Human CT55 sgRNA | GTCGGCCGTCCTCCCGTAGA |
| Human CT55 check F1 | GACCTGAGGGCTTCCCTTAC |
| Human CT55 check R1 | ATTGTTGAGGACTCGGGTTG |

**Supplementary Table 2: Primers for real-time PCR detection.**

| Primer | Sequence 5'---3' |
| --- | --- |
| IL-1β-M-Forward Primer  IL-1β-M-Reverse Primer  TNF-α-M-Forward Primer  TNF-α-M-Reverse Primer  Il6-M-Forward Primer  Il6-M-Reverse Primer  ccl2-M-Forward Primer  ccl2-M-Reverse Primer  COX2-M-Forward Primer  COX2-M-Reverse Primer  PCNA-M-Forward Primer  PCNA-M-Reverse Primer  β-catenin-M-Forward Primer  β-catenin-M-Reverse Primer  cyclinD1-M-Forward Primer  cyclinD1-M-Reverse Primer  TNF-α-H-Forward Primer  TNF-α-H-Reverse Primer  IκBα-H-Forward Primer  IκBα-H-Reverse Primer  cIAP2-H-Forward Primer  cIAP2-H-Reverse Primer  β-actin-M-Forward Primer  β-actin-M-Reverse Primer  β-actin-H-Forward Primer  β-actin-H-Reverse Primer  Ct55-M-Forward Primer  Ct55-M-Reverse Primer  CT55-H-Forward Primer  CT55-H- Reverse Primer | CCGTGGACCTTCCAGGATGA  GGGAACGTCACACACCAGCA  AGCCGATGGGTTGTACCTTG  ATAGCAAATCGGCTGACGGT  AGGATACCACTCCCAACAGACCT  CAAGTGCATCATCGTTGTTCATAC  TAAAAACCTGGATCGGAACCAAA  GCATTAGCTTCAGATTTACGGGT  TGCACTATGGTTACAAAAGCTGG  TCAGGAAGCTCCTTATTTCCCTT  TTGCACGTATATGCCGAGACC  GGTGAACAGGCTCATTCATCTCT  ATGGAGCCGGACAGAAAAGC  TGGGAGGTGTCAACATCTTCTT  GCGTACCCTGACACCAATCTC  ACTTGAAGTAAGATACGGAGGGC  TACTCCCAGGTCCTCTTCAAGG  TTGATGGCAGAGAGGAGGTTG  CGGGCTGAAGAAGGAGCGGC  ACGAGTCCCCGTCCTCGGTG  TCAA GTTCAAGCCAGTTACC  GACTCTGCATTTTCATCTCC  GTGACGTTGACATCCGTAAAGA  GCCGGACTCATCGTACTCC  GATCATTGCTCCTCCTGAGC  ACTCCTGCTTGCTGATCCAC  ATGCACCGGCTAATTTCCAGA  CCCTGTTTGTTTTGTAGTGAGGT  CAAGGTGACACCCAGTTGAC  CAACATCCACCTTGATTGCT |
